# Carbohydrate Recognition by an Architecturally Complex α-*N*-Acetylglucosaminidase from *Clostridium perfringens*

**DOI:** 10.1371/journal.pone.0033524

**Published:** 2012-03-27

**Authors:** Elizabeth Ficko-Blean, Christopher P. Stuart, Michael D. Suits, Melissa Cid, Matthew Tessier, Robert J. Woods, Alisdair B. Boraston

**Affiliations:** 1 Biochemistry and Microbiology, University of Victoria, Victoria, British Columbia, Canada; 2 Complex Carbohydrate Research Center, University of Georgia, Athens, Georgia, United States of America; 3 School of Chemistry, National University of Ireland, Galway, Ireland; Consejo Superior de Investigaciones Cientificas, Spain

## Abstract

CpGH89 is a large multimodular enzyme produced by the human and animal pathogen *Clostridium perfringens*. The catalytic activity of this *exo*-α-d-*N*-acetylglucosaminidase is directed towards a rare carbohydrate motif, *N*-acetyl-β-d-glucosamine-α-1,4-d-galactose, which is displayed on the class III mucins deep within the gastric mucosa. In addition to the family 89 glycoside hydrolase catalytic module this enzyme has six modules that share sequence similarity to the family 32 carbohydrate-binding modules (CBM32s), suggesting the enzyme has considerable capacity to adhere to carbohydrates. Here we suggest that two of the modules, CBM32-1 and CBM32-6, are not functional as carbohydrate-binding modules (CBMs) and demonstrate that three of the CBMs, CBM32-3, CBM32-4, and CBM32-5, are indeed capable of binding carbohydrates. CBM32-3 and CBM32-4 have a novel binding specificity for *N*-acetyl-β-d-glucosamine-α-1,4-d-galactose, which thus complements the specificity of the catalytic module. The X-ray crystal structure of CBM32-4 in complex with this disaccharide reveals a mode of recognition that is based primarily on accommodation of the unique bent shape of this sugar. In contrast, as revealed by a series of X-ray crystal structures and quantitative binding studies, CBM32-5 displays the structural and functional features of galactose binding that is commonly associated with CBM family 32. The functional CBM32s that CpGH89 contains suggest the possibility for multivalent binding events and the partitioning of this enzyme to highly specific regions within the gastrointestinal tract.

## Introduction

Mucins are heavily O-glycosylated glycoproteins that act to protect the epithelia from harmful bacteria by forming a biophysical barrier to infection as well as supporting innate and adaptive immunity [1]. A heavily hydrated and highly viscous protective mucosal layer can be found lining the surface of the major entry points to our body, including the eyes, the naso-pharynx, the genito-urinary tract and the gastrointestinal tract. Within the gastrointestinal tract the mucin layer can vary from 700 µm deep in the stomach to 150–300 µm deep in the small intestine [2]. Pathogens of the gastrointestinal tract, such as *Clostridium perfringens*, must find ways to subvert or somehow challenge this protective mucosal barrier in order to set up infection.

*C. perfringens'* niche environment is in the gut of animals, including humans, where it may reside harmlessly; however, infection with a pathogenic strain can cause gastroenteritis and, in serious cases, substantial intestinal tissue destruction associated with necrotic enteritis. Among the enzymes that *C. perfringens* employs to cope with the mucosal surface are the glycoside hydrolases, which have varying catalytic specificities that reflect the diversity in host glycans; these include, but are not limited to, neuraminidases (GH33)[3], [4], exo- and endo-β-*N*-acetylglucosaminidases (GH84 and GH85)[5], [6], [7], an endo-α-*N*-acetylgalactosaminidase (GH101)[8], [9], as well as CpGH89, which is an exo-α-*N*-acetylglucosaminidase [10], [11]. Due to the significant genome content of genes encoding carbohydrate-active enzymes with known or suspected specificity for complex glycans, such as those found on the mucosal surface, it has been postulated that these enzymes play an important role during colonization and/or infection. Indeed, enzymatic preparations of *C. perfringens*, in combination with mild acid hydrolysis, have previously been used to help partially “untangle” the complex carbohydrate surface lining the gut supporting the concept that the structure of gastrointestinal mucosa can be influenced by these bacterial factors [12].

Within the gastric mucosa there are two types of mucous cells, surface mucous cells and the deeper gland mucous cells, producing two different mucins which combine together to form a stratified surface mucous layer [13]. Class III mucins are produced normally by the gastric gland mucous cells, duodenal Brunner's gland mucous cells, and the mucous cells of the accessory glands of pancreaticobiliary tract but also in certain tissues exhibiting gastric metaplasia or adenocarcinoma [14]–[23]. The class III mucins, discharged by gland mucous cells in the gastric pits [13], are somewhat distinct in that they are specifically decorated with peripheral α-GlcNAc (α-*N-*acetyl-d-glucosamine) residues forming GlcNAc-α-1,4-Gal-β-R (*N*-acetyl-β-d-glucosamine-α-1,4-d-galactose) motifs [19], [22], [24]. The biological relevance of this carbohydrate motif is at present not clear; however, terminal α-linked GlcNAc has been implicated as a host defense mechanism against colonization of the gastric mucosa by *Helicobacter pylori* [25] by blocking production of CGL (cholesteryl-α-d-glucopyranoside), an important component of this bacterium's cell wall.

*C. perfringens* is unusual in its ability to process the GlcNAc-α-1,4-Gal motifs found in class III mucin. CpGH89 (EC 3.2.1.50, CPF_0859), also referred to as AgnC [11], is a family 89 α-*N-*acetyl-d-glucosaminidase that has been shown to specifically release terminal α-linked GlcNAc from the disaccharide GlcNAc-α-1,4-Gal and demonstrated to liberate GlcNAc from crude class III porcine gastric mucin [10], [11]. Using a *cpgh89* mutant of *C. perfringens* the activity of CpGH89 has been linked to the ability of *C. perfringens* to grow on mucin bearing this rare carbohydrate motif [11].

**Figure 1:**
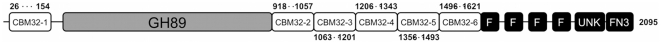
Schematic representation of the modular structure of CpGH89. CBM32 denotes family 32 carbohydrate-binding modules, GH89 represents the family 89 catalytic module, F denotes the predicted FIVAR (Found In Various Architectural Regions) modules and FN3 refers to a fibronectin type III domain. Modular boundaries used in this study are given above and below the schematic.

Two remarkable features of CpGH89 are its overall size (2095 amino acids) and its extensive multimodularity. Overall, the enzyme comprises a glycoside hydrolase family 89 (GH89) catalytic module, four FIVAR (found in various molecular architectures) modules, an unknown module, a C-terminal fibronectin type III-like (FN3-like) module, and six putative carbohydrate-binding modules (CBMs) (Figure 1). CBMs are generally defined as non-catalytic modules that bind carbohydrates and are found within the modular architectures of carbohydrate-active enzymes [26], thus distinguishing these modules from lectins and carbohydrate-specific antibodies. CBMs are presently classified into over 60 amino acid sequenced based families; the CBMs from CpGH89 all belong to CBM family 32, which is one of the most diverse CBM families [7].

Based on truncation studies of the enzyme and structural analyses of the N-terminal modules, the catalytic activity of the enzyme allowing it to release GlcNAc from class III mucin is attributed to its GH89 module [10], [11]. Similar truncation studies that focused solely on CBM32s 2 to 6 revealed one or more of these CBMs to be able to bind mucin [11]. Notably, constructs of CpGH89 lacking the three most C-terminal CBMs had reduced activity on mucin suggesting an important role for the CBMs in substrate recognition. Thus, CpGH89 possesses a complex multimodular architecture where the composite modules function together to efficiently act on components of mucin. Though it is clear that the CBMs are able to bind mucin what remains unknown is what carbohydrate motifs displayed on mucin, particularly the unique GlcNAc-α-1,4-Gal motif, may be recognized by the CBM32s and what the molecular bases of these interactions are. Here we address these questions through structural and functional analyses of the CBMs from CpGH89. Overall, these studies reveal the specificity of three of CBM32s and, through X-ray crystal structures, how two of the CBMs accommodate their ligands, which includes the first GlcNAc-α-1,4-Gal binding specificity for a protein other than an antibody.

## Results and Discussion

### Analysis of a galactose binding CBM

**Figure 2:**
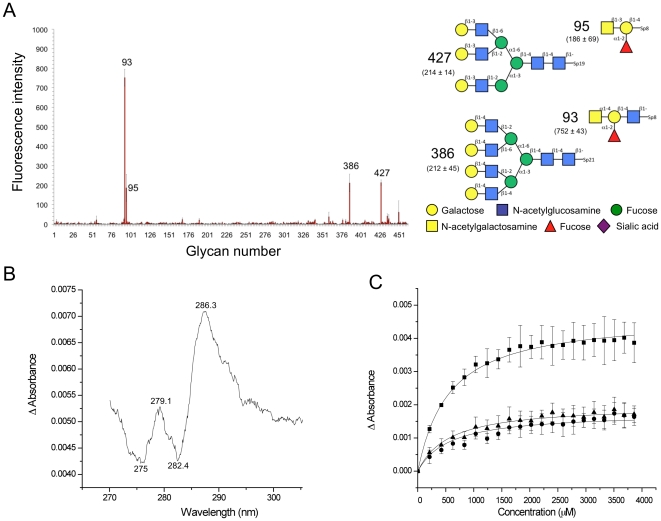
Analysis of carbohydrate binding by CBM32-5. (A) Glycan microarray analysis of carbohydrate binding by CBM32-5. The carbohydrates giving the most significant signals are numbered and their structures are shown schematically to the immediate right. The fluorescence intensities measure for the glycans are shown with the structural schematics. The reported error represents the standard error of the mean for quadruplicate measurements. A legend to symbols representing specific monosaccharides is provided. (B) UV difference spectrum upon CBM32-5 binding to excess galactose. Wavelengths of peaks and troughs are labeled. (C) Binding isotherm of CBM32-5 binding to galactose generated by UV difference titrations. The squares, triangles and circles represent the values for the peak to trough height differences for the wavelength pairs of 287.2/282.4 nm, 279.1/275.3 nm, and 279.1/282.4 nm, respectively. The error bars represent the standard deviations from triplicate independent titrations. The solid lines show the fits to a one-site binding model.

Of the six putative CBM32s in CpGH89 CBM32-5, the fifth CBM, has the highest similarity with modules known to have carbohydrate-binding function (∼43% amino acid sequence identity with the CBM32 from the large sialidase NanJ, also from *C. perfringens*). Furthermore, the strict conservation of residues involved in galactose recognition suggested that CBM32-5 belongs to the galactose binding group of family 32 CBMs [7], [27], [28]. CBM32-5 was initially screened for carbohydrate binding on glycan microarrays. Binding was generally quite weak; however, two galactose terminating N-glycans, one tri-antennary and the other tetra-antennary, gave significant binding signal (Figure 2A). Likewise, two glycans terminating with GalNAc, one α-1,4-linked and the other β-1,3-linked, also gave good signals. Though this did not conclusively single out a single carbohydrate ligand it is generally consistent with predictions of galactose specificity based on amino acid sequence similarity. This suggested binding to terminal galactose and GalNAc residues, which was used as a guide to quantitatively assess binding to carbohydrate ligands.

The addition of galactose or GalNAc to CBM32-5 perturbed the UV absorption of this protein in a manner consistent with the involvement of tyrosine residues in carbohydrate binding [29](Figure 2B). This signal was used in a quantitative manner to assess binding to a variety of carbohydrate ligands (Figure 2C and Table 1). The association constants of CBM32-5 binding to ligands containing galactose or GalNAc were in the range of 2–5×10^3^ M^−1^ (Figure 2B, 2C and Table 1), and thus quite weak, but of the same magnitude observed for other family 32 CBMs [3], [27], [30]–[32]. The CBM displayed little to no preference for either galactose or GalNAc and did not appear to significantly favor common disaccharide motifs that terminate in galactose or GalNAc over the monosaccharides (Table 1).

**Table 1:** Affinity of CBM32-5 for carbohydrates determined at 20°C in 20 mM Tris HCl, pH 8.0.

Carbohydrate	K_a_ (M^−1^)
d-galactose	1.69 (±0.05)×10^3^
d-GalNAc	5.01 (±0.64)×10^3^
Lactose (Gal-β-1,4-Glc)	2.40 (±0.58)×10^3^
Gal-β-1,3-GalNAc	3.77 (±0.34)×10^3^
GalNAc-β-1,3-Gal^a^	3.40 (±0.10)×10^3^

a the binding constant for GalNAc-β-1,3-Gal was determined by ITC; the remaining binding constants were determined by UV difference titrations.

**Figure 3:**
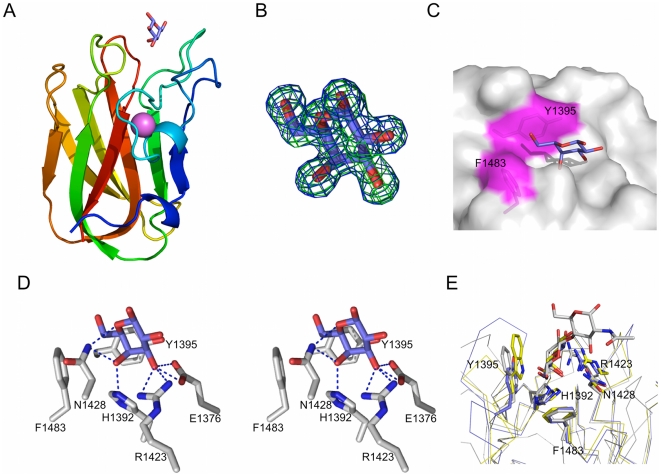
Structural analysis of the interaction between CBM32-5 and galactose. (A) A cartoon representation of the structure of CBM32-5 bound to galactose (blue sticks) determined by X-ray crystallography to 1.55 Å resolution. The bound calcium atom is shown as a pink sphere. (B) Electron density for galactose within the binding site of CBM32-5. Electron density maps are maximum-likelihood/σ_A_ [59] -weighted 2*F*_obs_-*F*_calc_ contoured at 1 σ (both maps at 0.45 e^−^/Å^3^) produced by refinements prior to modeling the sugar (green) and with the monosaccharide included (blue). (C) Surface representation of the CBM32-5 binding site with the bound galactose shown as blue sticks. The aromatic amino acids providing the hydrophobic binding platform are shown as sticks and labeled while the surface they contribute to the active site is coloured magenta. (D) Divergent stereo view of the key interactions between the binding site of CBM32-5 and galactose. Hydrogen bonds are shown as dashed black lines. (E) Comparison of the binding site of CBM32-5 (blue) with the CBM32 from *C. perfringens* GH84C (grey; PDB code 2J1E) and the CBM32 from *C. perfringens* NanJ (yellow; PDB code 2V72) reveals the canonical galactose-binding site. Conserved amino acid side chains and bound carbohydrates are shown as sticks.

**Figure 4:**
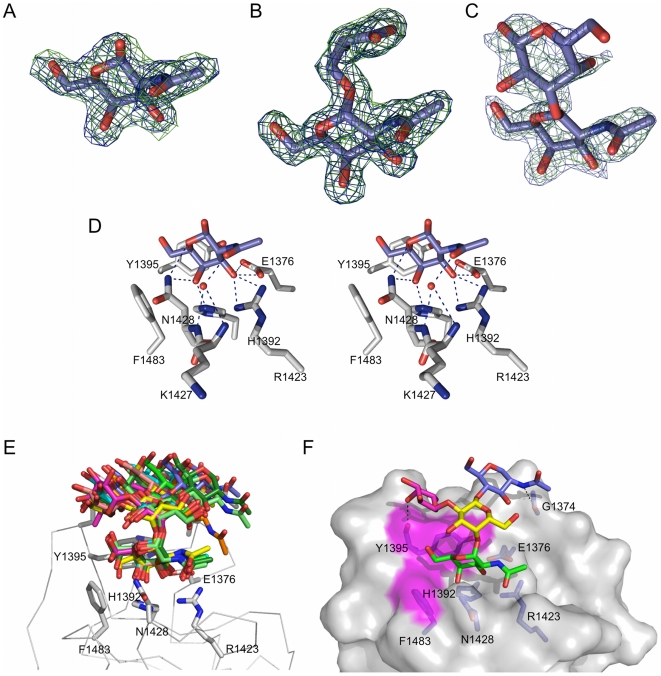
Structural analysis of CBM32-5 with additional carbohydrates. (A) Electron density for GalNAc shown as maximum-likelihood/σ_A_ [59] -weighted 2*F*_obs_-*F*_calc_ maps contoured at 1 σ (both maps at 0.31 e^−^/Å^3^) produced by refinements prior to modeling the sugar (green) and with the sugar included (blue). (B) Electron density for serinyl-Tn antigen shown as maximum-likelihood/σ_A_ [59] -weighted 2*F*_obs_-*F*_calc_ maps contoured at 1 σ (both maps at 0.45 e^−^/Å^3^) produced by refinements prior to modeling the sugar (green) and with the sugar included (blue). (C) Electron density for GalNAc-β-1,3-galactose shown as maximum-likelihood/σ_A_ [59] -weighted 2*F*_obs_-*F*_calc_ maps contoured at 0.8 σ (both maps at 0.34 e^−^/Å^3^) produced by refinements prior to modeling the sugar (green) and with the sugar included (blue). (D) Divergent stereo view of the key interactions between the binding site of CBM32-5 and GalNAc. This also represents the mode of interaction between the CBM and the serinyl-Tn antigen and GalNAc-β-1,3-galactose, which all have identical hydrogen bonding patters. Hydrogen bonds are shown as dashed black lines. (E) Models of CBM32-5 in complex with GalNAc-α-1,4(Fuc-α-1,2)-Gal-β-1,4-GlcNAc-OMe produced by molecular dynamics simulations. An ensemble of ten energy minimized models is given with each model representing a group of energetically similar models. Relevant residues in the binding site are shown as grey sticks with the backbone of the protein shown as a Cα-ribbon. (F) A surface representation of the lowest energy model of CBM32-5 bound to the tetrasaccharide (GalNAc is shown in green, fucose in pink, galactose in yellow, and GlcNAc in blue). The surfaces contributed by Y1395 and F1483 are shown in magenta and additional hydrogen bonds made outside of the primary galactose binding site shown as dashed lines.

The structural basis for what appears to be a general selectivity for terminal galactose residues was examined by determining the X-ray crystal structure of CBM32-5 in complex with carbohydrate. The 1.55 Å resolution structure of the CBM binding galactose revealed the β-sandwich fold with structural metal ion, in this case modeled as a Ca^2+^, which is common to the family (Figure 3A). The galactose residue was well-ordered in the crystal structure providing clear electron density (Figure 3B). The site accommodating this carbohydrate is a shallow cleft marked by two solvent exposed aromatic side chains, F1483 and Y1395 (Figure 3C), which is present in the loops at the edges of the β-sandwich (Figure 3A). The C6-OH group of galactose fits into a corner of the binding site made up by F1483 and Y1395, whose aromatic rings are at nearly right angles to one another (Figure 3C and 3D). A series of hydrogen bonds involve the side chains of four amino acids in the carbohydrate-binding site (Figure 3D). With the exception of E1376, which makes hydrogen bonds with the C3 hydroxyl group of galactose, all of the interactions are highly conserved with other known galactose binding CBMs (Figure 3E). Indeed, the interactions made by the five residues H1392, Y1395, R1423, N1428, and F1483 make up the canonical galactose-binding motif in the family 32 CBMs [7], [27], [32]. CBM32-5, therefore, possesses a galactose-binding site; however, it is also capable of binding GalNAc equally well. Furthermore, the analysis of the CBM32 from NagJ, indicated that the recognition of longer glycans by CBM32s can involve additional subsites [27]. The structures of CBM32-5 in complex with other potentially biologically relevant ligands, GalNAc, the Tn-antigen, and GalNAc-β-1,3-Gal (Figure 4A, 4B and 4C) show the recognition of terminal GalNAc residues to be identical to that of galactose, with the addition of a water mediated hydrogen bond involving the acetamido group of the carbohydrate and the backbone nitrogens of K1427 and N1428 (Figure 4D). This limited additional interaction appears to provide little to no favorable energy to binding. Likewise, the galactose of the GalNAc-β-1,3-Gal extended away from the protein surface and made no interactions with the protein, which is consistent with the lack of improved binding for this disaccharide over GalNAc. The same observation was made for the serinyl group of the Tn-antigen, even though the serine is α-linked to GalNAc. Modeling other common α-linked carbohydrates, such as Gal-α-1,3-Gal, based on the Tn-antigen complex suggested that these additional residues also extend out into solvent with no capacity to make additional interactions with the protein (not shown).

The crystallography results suggest that CBM32-5 is relatively promiscuous in that it requires only a terminal galactose or GalNAc residue with little preference for the sugar that precedes it. The glycan microarray results, however, suggested a strong interaction with a unique carbohydrate, GalNAc-α-1,4(Fuc-α-1,2)-Gal-β-1,4-GlcNAc. This interaction was reproducible on glycan microarrays, even when using CBM that was directly labeled by chemically coupling the fluorophore to primary amines on the CBM (not shown). To our knowledge, this glycan has not been identified in any mammalian tissues; however, this synthetic carbohydrate was clearly the top ligand from the array analysis suggesting that an analysis of the interaction of CBM32-5 with this carbohydrate may provide insight into the recognition of more complex but as yet unstudied glycans. A molecular dynamics approach was used to study the potential interaction of GalNAc-α-1,4(Fuc-α-1,2)-Gal-β-1,4-GlcNAc-OMe with CBM32-5. The resulting analysis gave an ensemble of ten structures with each structure representing a group of similar, energy-minimized structures (Figure 4E). Overall, the carbohydrate in the ten structures adopts an array of potential conformations, though the terminal GalNAc residue and the preceding Gal residue are somewhat constrained in their positions. A representative of the lowest energy group of models shows the carbohydrate to adopt a conformation that, by virtue of the bent conformation imparted by the α-1,4-linkage between the GalNAc and Gal, bends around Y1395 and allows the reducing-end portion of the glycan to rest against the protein surface with only a very small number of additional hydrogen bonds made (Figure 4F). Free energy decomposition shows the increased affinity of this ligand for CBM32-5 results from the increased van der Waals and non-polar solvation interactions that is imparted by the complementary interacting surface areas of this unique carbohydrate ligand and the CBM surface. This interaction is specifically enhanced by interactions between the fucosyl residue and residues Y1395 and N1396 of CBM32-5 (Figure S1). Though GalNAc-α-1,4(Fuc-α-1,2)-Gal-β-1,4-GlcNAc may not be a biologically relevant ligand for CBM32-5 its mode of interaction with this CBM suggests that other high affinity ligands, perhaps not represented on the carbohydrate microarrays, may be possible provided they adopt a conformation that maximizes the interacting surface areas.

### Carbohydrate-binding modules with unique specificity

**Figure 5:**
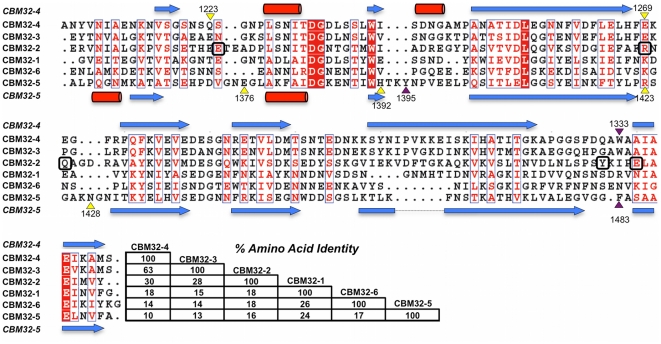
Amino acid sequence comparison of the CBM32 modules from CpGH89. The secondary structure is shown above (CBM32-4) and below (CBM32-5) with arrows representing β-strands and cylinders α-helices. The purple and yellow triangles above and below the sequences indicate the aromatic and hydrogen bonding residues, respectively, that are involved in carbohydrate binding by CBM32-4 (top) and CBM32-5 (bottom). Numbers with the triangles indicate the residue number. Residues in CBM32-2 that are highlighted by boxes are those present in the putative binding site of this module.

**Figure 6:**
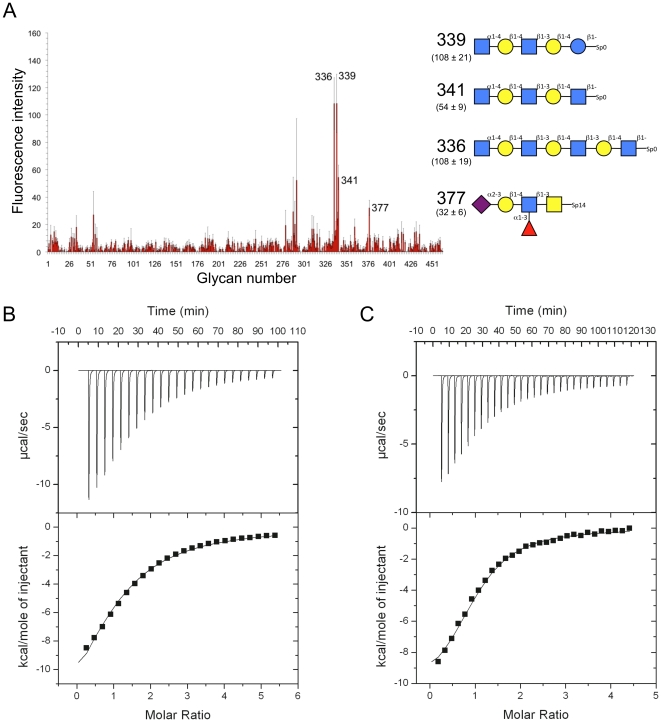
Analysis of carbohydrate binding by CBM32-3 and CBM32-4. (A) Glycan microarray analysis of carbohydrate binding by CBM32-3. The carbohydrates giving the most significant signals are numbered and their structures are shown schematically to the immediate right. The fluorescence intensities measured for the glycans are shown with the structural schematics. The reported error represents the standard error of the mean for quadruplicate measurements. The symbols representing specific monosaccharides are the same as those given for Figure 2A. (B) and (C) Representative isothermal calorimetry titrations of CBM32-4 and CBM32-3, respectively, binding to GlcNAc-α-1,4-Gal. Top portions of each panel show the raw power data while the bottom portions show the integrated and heat of dilution corrected data. Solid lines show the non-linear curve fits to a one site binding model.

Though CpGH89 has at least one functional CBM its specificity (i.e. galactose and GalNAc) is clearly mismatched with the specificity of the catalytic module. Furthermore, this CBM is an outlier among the CpGH89 CBMs as it has higher amino acid sequence identity with CBMs from other enzymes than it does with the remaining CBMs from CpGH89. In contrast, CBM32-2, CBM32-3, and CBM32-4 form a distinct cluster in the phylogenetic analysis of the CBM32 family [7]. Indeed, CBM32-3 and CBM32-4 share 63% amino acid sequence identity and CBM32-2 has ∼30% amino acid identity with these two CBMs (Figure 5). These putative CBMs have very low amino acid sequence identity with CBM32-5 and other known CBM32s suggesting they may represent a new functional class of CBM32s. Isolated CBM32-2, CBM32-3, and CBM32-4 were screened for binding on the glycan microarrays. CBM32-3 gave statistically meaningful binding (i.e. signal with standard errors of the mean that indicated significant binding above background) with the top hits terminating in GlcNAc-α-1,4-Gal (Figure 6A). Unfortunately, the results for CBM32-2 and CBM32-4 were inconclusive; however, the high amino acid sequence similarity between CBM32-3 and CBM32-4 suggested that both CBMs may have the same ligand, GlcNAc-α-1,4-Gal. Indeed, using ITC, the association constant of CBM32-4 for GlcNAc-α-1,4-Gal was determined to be 1.38 (±0.08)×10^4^ M^−1^ thus showing this to be a relatively strong interaction for a family 32 CBM (Figure 6B). The titration of GlcNAc-α-1,4-Gal into CBM32-3 also produced a binding isotherm consistent with carbohydrate binding and the association constant was determined to be 2.64 (±0.64)×10^4^ M^−1^ (Figure 6C). Thus, both CBM32-3 and CBM32-4 appear to have binding specificity for GlcNAc-α-1,4-Gal, which is complementary to the specificity of the catalytic module.

**Figure 7:**
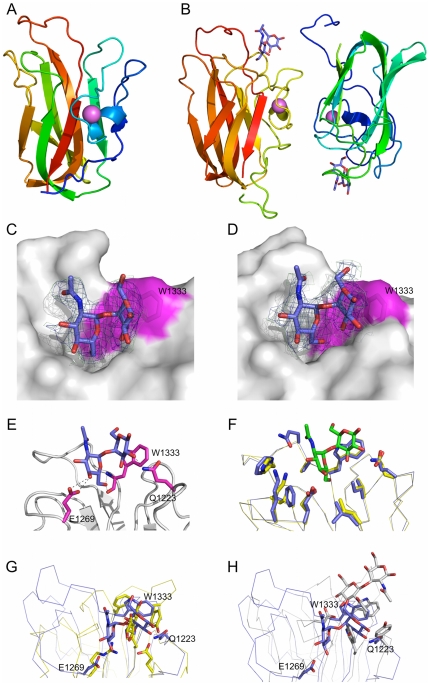
Structural analysis of the interaction between CBM32-4 and GlcNAc-α-1,4-Gal. (A) A cartoon representation of the structure of seleno-methionine labeled CBM32-4 determined by X-ray crystallography to 1.55 Å resolution. The bound calcium atom is shown as a pink sphere. (B) A cartoon representation of the dimer of CBM32-4 in complex with GlcNAc-α-1,4-Gal determined to 2.8 Å resolution. The bound calcium atom is shown as a pink sphere and the carbohydrate as blue sticks. (C) and (D) Electron density for GlcNAc-α-1,4-Gal in the binding sites of each monomer of CBM32-4 in the asymmetric unit (panel C shows molecule A and panel D shows molecule B). In both panels the electron density is shown as maximum-likelihood/σ_A_ [59] -weighted 2*F*_obs_-*F*_calc_ maps contoured at 1 σ (all maps at 0.39 e^−^/Å^3^) produced by refinements prior to modeling the sugar (green) and with the sugar included (blue). Protein is shown as solvent accessible surface with the surface contributed by W1333 shown and labeled) colored magenta. (E) Key interactions between the binding site of CBM32-4 and the disaccharide as represented by monomer A in the asymmetric unit. (F) An overlay of CBM42-4 in complex with the disaccharide (blue with green carbohydrate) and a Phyre2 [58] generated homology model of CBM32-3 (yellow) showing the conservation of residues lining the binding site. (G) and (H) Superposition of CBM32-4 in complex with the disaccharide (blue in both panels) with CBM32-5 in complex with galactose (yellow molecule in panel G) and with the GlcNAc binding CBM32 from *C. perfringens* NagH (PDB code 2W1U; grey molecule in panel H). Relevant residues involved in carbohydrate recognition are shown as sticks. Only residues in CBM32-4 are labeled.

The ability of CBM32-3 and CBM32-4 to bind the GlcNAc-α-1,4-Gal is unique among non-catalytic carbohydrate binding proteins prompting the study of the molecular basis of this interaction. Of the two CBMs, crystals were only obtained of CBM32-4. The structure of seleno-methionine labeled CBM32-4 was determined by single anomalous dispersion to 1.55 Å resolution. This CBM adopts a β-sandwich fold with conserved structural metal ion, modeled as a calcium atom, which is similar to that of CBM32-5 (root mean square deviation of 1.9 Å over 112 matched Cα) (Figure 7A). CBM32-4 was co-crystallized with GlcNAc-α-1,4-Gal and this structure determined to 2.8 Å resolution (Figure 7B). Both molecules of CBM32-4 in the asymmetric unit had bound disaccharide as revealed by clear electron density for the sugar located in the loops at the edges of the β-sandwich core (Figure 7B, C, and D). CBM32-4 accommodates the disaccharide in a shallow depression; the sugar, with its bent conformation, lies on edge in the depression with the B-face of the galactose residue pushed up against the planar surface of the W1333 side chain. Though there are no aromatic residues present on the adjacent wall of the binding site, it is at roughly right angles to the plane of the W1333 side chain and thus well positioned to pack against the A-face of the GlcNAc residue. Markedly few hydrogen bonds are made between the sugar and binding site suggesting that binding and specificity for this disaccharide is driven primarily by hydrophobic and van der Waals forces and accommodation of the unique carbohydrate conformation. O1 of the galactose is completely exposed and oriented out into the bulk solvent illustrating how the CBM might tolerate extensions on the reducing end of the GlcNAc-α-1,4-Gal motif, which is consistent with binding to the glycan microarrays and to the recognition of the motif as it would naturally be displayed at the termini of glycans on mucin. The O3 and O4 groups on the terminal GlcNAc, though solvent exposed, lie very close to the protein surface. It is unclear whether modification to these could be tolerated by the CBM, thereby allowing it to recognize internal GlcNAc-α-1,4-Gal motifs, but the proximity to the protein surface and steric clashes that would likely ensue suggests that this is unlikely. The C6 hydroxyl group is buried in the base of the binding site and thus extension with additional sugar residues would not be tolerated.

CBM32-3 was recalcitrant to crystallization preventing structural analysis by X-ray crystallography and direct examination of its interaction with carbohydrate; however, the main residues involved in GlcNAc-α-1,4-Gal recognition by CBM32-4 are conserved in CBM32-3 (Figure 5). Taking further advantage of the high amino acid sequence identity of the two CBMs, a homology model of CBM32-3 was constructed; this revealed not only conservation of the primary binding site residues but also the majority of the residues lining the binding site (Figure 7F), indicating that the mode of carbohydrate recognition by CBM32-3 is likely extremely similar to that of CBM32-4.

To date, the structural analysis of family 32 CBMs found in carbohydrate-active enzymes has revealed two subtypes of CBMs within the family: the ‘canonical’ galactose binding CBM32s, such as CBM32-5, and the unique GlcNAc binding CBM32 as represented by the CBM from NagH, NagHCBM32-2 [31]. A comparison of the amino acids involved in ligand binding from CBM32-4 with the binding sites of both of these CBM32 subtypes shows them to have no similarities in carbohydrate recognition beyond the general placement of the active sites (Figure 7G and 7H). Thus, the GlcNAc-α-1,4-Gal binding CBMs, CBM32-3 and CBM32-4, represent a new mode of carbohydrate recognition by the CBM32s and continue to highlight the diversity within this family of CBMs.

**Figure 8:**
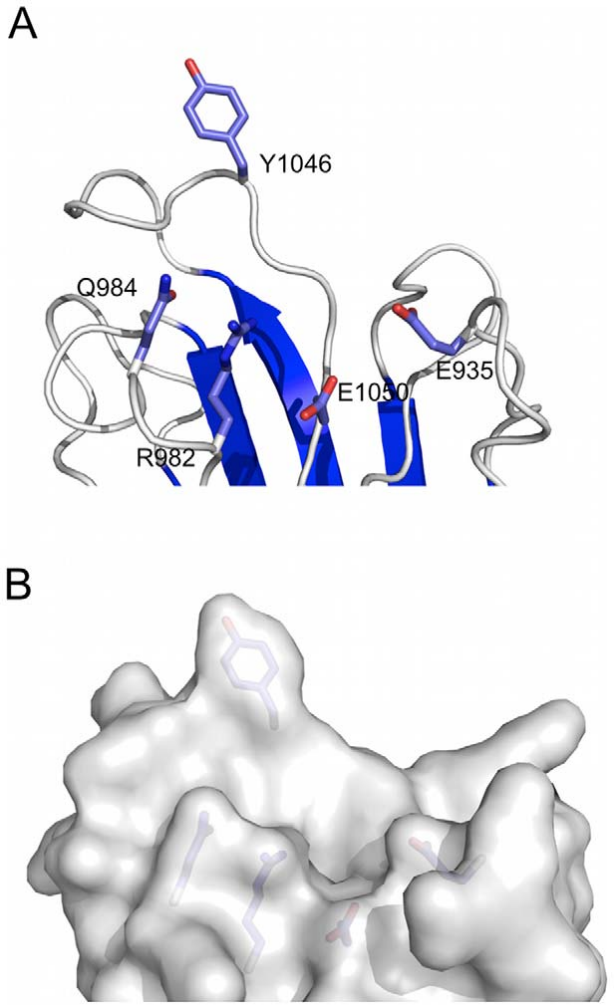
Homology model of CBM32-2 generated with Phyre2 [58]. (A) Architecture of the putative carbohydrate binding site with residues possibly involved in sugar recognition shown as sticks. (B) Solvent accessible surface of the putative binding site revealing its contours.

Glycan microarray binding experiments with CBM32-2 were inconclusive, as were other low-throughput experiments to identify potential ligands, and attempts at crystallization did not yield crystals of sufficient quality for structure determination. To provide some insight into the potential capacity of this module to interact with carbohydrate a homology model based on the structure of CBM32-4 was constructed. Though the residues in CBM32-4 that impart carbohydrate binding function are not conserved with CBM32-2 (Figure 5) the model reveals a pocket in the protein surface located in loops that usually contain the binding sites of CBM32s (Figure 8A and 8B). This pocket contains a solvent exposed aromatic amino acid, Y1046, and a series of exposed planar polar amino acid side chains (Figure 8B). These features are generally consistent with the properties of carbohydrate binding sites in CBMs, suggesting that this module is indeed capable of recognizing an as yet unidentified sugar.

### CBM32-1 and CBM32-6 appear to lack carbohydrate-binding function

**Figure 9:**
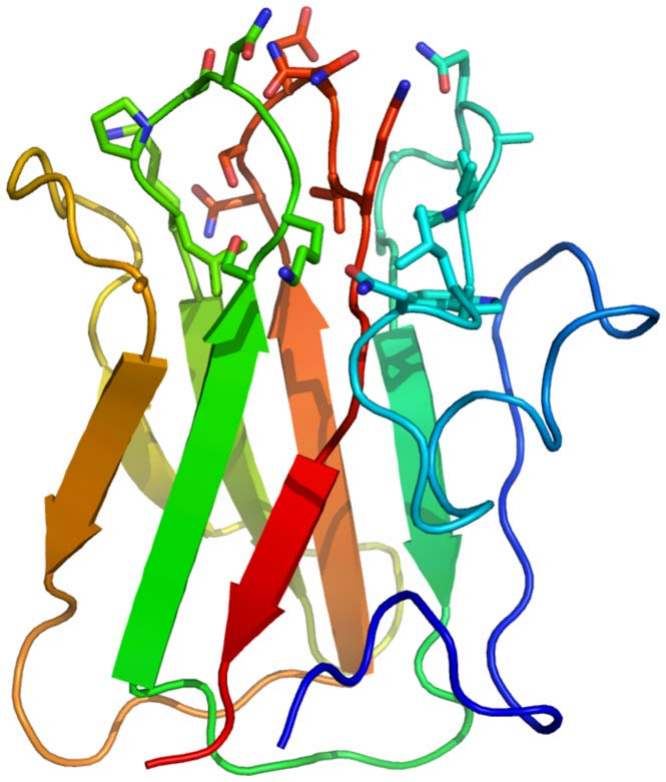
Structural analysis of CBM32-6. A cartoon representation of the structure of CBM32-6 determined by X-ray crystallography to 1.55 Å resolution. Amino acid side chains found in what is normally the carbohydrate-binding site of family 32 CBMs are shown in stick representation. This reveals the lack of side chains normally associated with carbohydrate binding, particularly a lack of aromatic amino acid side chains.

Despite the observation that CBM32-1 and CBM32-6 display only 26% amino acid identity (Figure 5) they cluster together in a phylogenetic analysis of CBM32 modules indicating that they are more closely related to one another than to other putative CBMs [7]. Qualitative UV difference scans on CBM32-1 and CBM32-6 did not suggest binding to any simple monosaccharides (galactose, GalNAc, mannose, sialic acid, GlcNAc or glucose). CBM32-1 was also screened on glycan microarrays but significant binding was not detected. The structure of CBM32-6 was determined to 1.55 Å resolution using SAD and seleno-methionine substituted protein (not shown). This structure compared with CBM32-1, previously determined as part of a construct including the catalytic module [10], gave a root mean square deviation of 1.8 Å over 119 Cα atoms. Neither CBM32-1 nor CBM32-6 have any exposed aromatics in the region of the protein known to contain the binding sites in CBM32 proteins (Figure 9). Furthermore, a more thorough analysis of the surface residues of CBM32-1 and CBM32-6 showed them both to lack features consistent with carbohydrate binding sites. This observation, along with the lack of experimental support for carbohydrate binding, suggest that CBM32-1 and CBM32-6 do not function as CBMs, which perhaps explains their somewhat outlying position in the phylogenetic analysis of CBM32 modules [7].

### The modular diversity of CpGH89 and its implications

In order to colonize the gastrointestinal tract organisms must first infiltrate the mucosal surface. For example, the secreted mucosal surfaces of the colon are comprised of mainly Muc2, which forms both the thick outer mucous layer, that plays host to many commensal microbes, and the thin inner mucous layer that is impervious to bacteria [33], [34]. GlcNAc-α-1,4-Gal is displayed by the deeper gastric-type mucosal class III mucins, Muc5Ac and Muc6 [19] and the catalytic activity of CpGH89 is directed at this specific carbohydrate structure. Furthermore, two of the CBMs in this enzyme, CBM32-3 and CBM32-4, have evolved binding specificity complementary to the catalytic specificity. In a manner consistent with the generally proposed role of CBMs [26], CBM32-3 and CBM32-4 likely direct the enzyme to the secreted class III mucins within the deep mucosa of the stomach and duodenum, and in doing so promote substrate degradation by the catalytic module. The presence of two CBMs with the same specificities indicate the potential for a multivalent interaction, thereby increasing the overall apparent affinity of the enzyme for regions that display clusters of the GlcNAc-α-1,4-Gal motif.

Of the six CBM32-like modules that CpGH89 possesses two do not appear to bind carbohydrate (their functions, if they have any, remain unknown), one has putative carbohydrate-binding function (CBM32-2), and the remaining three clearly have carbohydrate-binding function (CBM32-3, CBM32-4 and CBM32-5). The specificity of CBM32-5 appears to be primarily for terminal galactose and GalNAc residues and thus does not match the substrate preference of the catalytic module. Such mismatching between CBMs and their cognate catalytic modules is not unusual with *C. perfringens* glycoside hydrolases [3], [27]. The biological reason for the presence of the mismatched CBMs remains speculative; however, it has been postulated that the presence of such CBMs may allow the enzyme to remain adhered to carbohydrate rich surfaces after the catalytic module has begun processing the substrate. For example, after hydrolysis of the GlcNAc-α-1,4-Gal substrate by the catalytic module of CpGH89 the remaining terminal sugar is a galactose residue and thus a potential ligand of CBM32-5. There then exists the potential for multivalent interactions involving heterogeneous clusters of ligands, such as combinations of the GlcNAc-α-1,4-Gal motif and terminal galactose and GalNAc residues. Alternatively, it has been hypothesized that the majority of the *C. perfringens* glycoside hydrolases, including CpGH89, are either covalently or non-covalently associated with the bacterial surface [6]. Thus, though the intrinsic affinity of a single CBM32-5 module for terminal galactose residues is quite low and on its own would be unlikely to mediate significant adherence of soluble CpGH89 to terminal galactose residues, the possible context of bacterial surface association of the entire enzyme creates further potential for avid binding.

Overall, the presence of at least three functional CBMs in CpGH89, with a fourth likely, imparts diversity in the ability of this enzyme to recognize carbohydrate substructures and potential for increased affinity through multivalent interactions. As a secreted enzyme this capability would enhance the overall association of the enzyme with class III mucins. In the possible case that CpGH89 is immobilized on the bacterial cell-surface the enzyme's capacity to bind carbohydrate would impart considerable carbohydrate-adhesive capacity to the bacterium thus promote the tight interaction of this bacterium with its host.

## Materials and Methods

### Cloning, protein production and purification

Gene fragments encoding desired CBMs from CpGH89 (locus tag CPF_0859) were PCR amplified from *C. perfringens* ATCC 13124 genomic DNA using oligonucleotide primers (see Table 2) with engineered 5′ and 3′ NheI and XhoI restriction endonuclease sites, respectively, incorporated into the ends of the primers. The following gene fragments were cloned into pET28a(+) through standard molecular biology procedures: CBM32-1 (nucleotides 76–462), CBM32-2 (nucleotides 2752–3171), CBM32-3 (nucleotides 3187–3603), CBM32-4 (nucleotides 3616–4029), CBM32-5 (nucleotides 4066–4479), CBM32-6 (nucleotides 4486–4863). All of the resulting gene fusions encoded an N-terminal six-histidine tag fused to the protein of interest by an intervening thrombin protease cleavage site. Bidirectional DNA sequencing was used to verify the fidelity of each construct.

**Table 2:** Oligonucleotide primers used for amplification and cloning.

Oligonucleotide	Sequence	Used to amplify and clone
CBM32-1F	CAT ATG GCT AGC GGT GTT GAA ATT ACG GAA G	CBM32-1
CBM32-2F	CAT ATG GCT AGC GAA AGA GTT AAT ATT GCT	CBM32-2
CBM32-3F	CAT ATG GCT AGC GAA GAT GAG TAT ACT AAC G	CBM32-3
CBM32-4F	CAT ATG GCT AGC GCT AAT TAT GTA AAT ATA G	CBM32-4
CBM32-5F	CAT ATG GCT AGC GCA TTA CCT CAA GGA AAT	CBM32-5
CBM32-6F	CAT ATG GCT AGC GAA AAC CTA GCT ATG AAA G	CBM32-6
CBM32-1R	GAA TTC CTC GAG TTA ACC AAA TAC ATT TAT TTC	CBM32-1
CBM32-2R	GAA TTC CTC GAG TTA ATA TAC CAT TAT TTC TGC	CBM32-2
CBM32-3R	GAA TTC CTC GAG TTA TGA CAT GGC CTT TAC TTC	CBM32-3
CBM32-4R	GAA TTC CTC GAG TTA ACT CAT AGC TTT AAT TTC	CBM32-4
CBM32-5R	GAA TTC CTC GAG TTA TGC AAA TAC ATT TAA TTC	CBM32-5
CBM32-6R	GAA TTC CTC GAG TTA TCC TTT ATA AAT TTT GAT	CBM32-6

All of the proteins were produced recombinantly in *E. coli* BL21(DE3) and purified by immobilized metal affinity chromatography and size exclusion chromatography (SEC) using methodologies described in detail previously [5]. Seleno-methionine-labeled CBM32-4 and CBM32-6 was produced as above using *E. coli* B834 (DE3) as the expression strain (Novagen). The media containing seleno-methionine was prepared according to the instructions of the manufacturer (Athena Enzyme). Protein concentrations were determined at 280 nm using calculated extinction coefficients [35] as follows: CBM32-1, 20340 M^−1^ cm^−1^; CBM32-2, 17780 M^−1^ cm^−1^; CBM32-3, 13940 M^−1^ cm^−1^; CBM32-4, 15220 M^−1^ cm^−1^; CBM32-5, 16500 M^−1^ cm^−1^; CBM32-6, 15220 M^−1^ cm^−1^.

### Glycan microarray screening

Glycan microarray screening was performed by Core H of the Consortium for Functional Glycomics (www.functionalglycomics.org/). CBMs were labeled by coupling to Alexa Fluor® 488 labeled streptavidin *via* a biotin-NTA:Ni^2+^ linker using methods identical to those described previously [36]. Labeled proteins were desalted using PD-10 columns (GE Healthcare) and used to probe the printed glycan arrays according to the standard procedures of Core H of the Consortium for Functional Glycomics.

### Binding studies

Qualitative UV difference scans were performed using methods identical to those described previously [31]. Quantitative UV difference titrations were also performed using methods already described [27]. The concentration of protein used for the titrations was 31.5 µM in 20 mM Tris-HCl pH 8.0. The concentrations of carbohydrate stocks used to titrate into protein varied between ∼40 mM and 45 mM and were prepared by mass in 20 mM Tris-HCl pH 8.0. Experiments were performed at 25°C in triplicate.

Isothermal Titration Calorimetry was performed as described previously using a VP-ITC (MicroCal, Northampton, MA)[27]. Proteins were filtered and degassed prior to use. Carbohydrate solutions were prepared by mass in buffer saved from dialysis of the appropriate protein. These solutions were also filtered and degassed prior to use. The proteins concentrations used varied from ∼100 µM to ∼550 µM. However, in no case could a protein concentration be used that exceeded the K_d_ by more than five-fold (i.e. C-values were less than 5), thus, data was fit with a single binding site model using MicroCal Origin software (version 7.0) with the stoichiometry (*n*-value) fixed at 1. Experiments using CBM32-5 were performed in 20 mM Tris-HCl, pH 8.0, and those with CBM32-3 and CBM32-4 in 50 mM HEPES, pH 7.5. Experiments were performed at 25°C in triplicate.

### Crystallization

Prior to crystallization, CBMs generally required overnight treatment with thrombin followed by re-purification by SEC to remove the 6-histidine tag. The complex of CBM32-4 with GlcNAc-α-1,4-Gal, however, was obtained with protein still having the 6-histidine tag. All crystallization experiments were performed at 18°C using the hanging drop vapour diffusion method.

Seleno-methionine labeled CBM32-4 at 15 mg/ml crystallized in 0.2 M KSCN, 22% polyethylene glycol (PEG) 3350, 0.1 M Tris-HCL pH 7.5. 20% ethylene glycol in crystallization solution was used as a cryoprotectant. Unlabeled CBM32-4 (20 mg/ml) in complex with GlcNAc-α-1,4-Gal (at 2 mM) crystallized in 0.1 M ZnOAc, 0.1 M Bicine pH 8.0, 18% PEG 3350, 4 mM CrCl; 20% ethylene glycol in this crystallization solution was used as a cryoprotectant.

All crystals of CBM32-5 were obtained using the protein at 20 mg/ml. Complexes were obtained by co-crystallization of the protein with the carbohydrate under the following conditions: the galactose (10 mM) and GalNAc (10 mM) complexes crystallized in 0.1 M Bis-Tris pH 5.5, 20% PEG 4000, and 0.2 M LiSO_4_; the GalNAc-β-1-3Gal (10 mM) complex crystallized in 0.1 M NaCitrate pH 5.6, 20% PEG 3350, and 0.2 M MgOAc; the Tn Antigen [10 mM; *N*-acetyl-α-d-galactosaminyl-1-O-serine (V-labs)] complex crystallized in 0.1 M NaCitrate pH 5.6, 20% PEG 3350, and 0.1 M ZnOAcetate. In all cases the crystals were cryoprotected using the crystallization solution supplemented with 15% glycerol.

Seleno-methionine labeled CBM32-6 (20 mg/ml) was crystallized in 0.1 M Bis-Tris pH 6.5, 29% PEG 3350, 0.05 M CaCl2 and 20% ethylene glycol in crystallization solution was used for cryoprotection.

### Data collection, Structure Solution and Refinement

Diffraction data were collected at 100 K at the National Synchrotron Light Source (NSLS) beamline X8-C, the Stanford Synchrotron Radiation Laboratories (SSRL) beamline BL 9-2, or a home source comprising a Rigaku R-AXIS IV++ area detector coupled to a MM-002 X-ray generator with Osmic “blue” optics and Oxford Cryostream 700 as indicated in Tables 3 and 4. Data were processed using d*trek or MOSFLM [37], [38].

**Table 3:** X-ray data collection and model refinement statistics for CBM32-5.

*Data collection statistics*	CBM32-5galactose	CBM32-5galNAc	CBM32-5TnAg	CBM32-5galNac-β-1,3-gal
Wavelength	1.5418	1.5418	1.5418	1.5418
Beamline	MM-002	MM-002	MM-002	MM-002
Space group	C2	C2	P2_1_2_1_2_1_	P2_1_2_1_2_1_
Resolution	20.00-1.55 (1.59-1.55)	30.00-1.90 (1.95-1.90)	20.00-1.70 (1.74-1.70)	20.00-1.75 (1.80-1.75)
Cell dimensionα, β, γ (Å)	65.27, 38.32, 53.8890.00 90.64 90.00	65.82, 37.27, 57.4190.00,103.51,90.00	31.80, 59.26, 67.4390.00, 90.00, 90.00	33.80, 56.31, 70.6690.00, 90.00, 90.00
*R_merge_*	0.061 (0.0244)	0.067 (0.323)	0.059 (0.314)	0.064 (0.378)
Completeness (%)	99.6 (99.5)	99.3 (97.8)	97.5 (95.2)	97.9 (98.0)
*<I/σI>*	13.0 (2.1)	11.6 (3.9)	12.0 (3.5)	10.7 (3.2)
Redundancy	3.1 (2.5)	6.1 (6.0)	4.3 (3.8)	4.4 (4.1)
Total reflections	66379	66214	61394	63956
Unique reflections	21421	10778	14287	14448
*Refinement statistics*				
*R* (%)	18.6	20.4	18.8	19.9
*R_free_* (%)	22.4	26.5	22.2	24.6
RMSD				
Bond lengths (Å)	0.013	0.014	0.012	0.015
Bond angles (°)	1.415	1.380	1.801	1.716
Average *B*-factors (Å^2^)				
Protein Chain	13.6	28.3	16.4	24.5
Water molecules	28.7	35.5	31.4	33.9
Ligand molecules	15.3	29.0	17.6	58.5
Number of atoms				
Protein atoms Chain A	1081	1076	1077	1063
Water molecules	273	141	227	155
Ligand molecules	12	15	21	26
Ramachandran statistics				
Most favored (%)	97.9	96.5	96.4	99.3
Additional allowed (%)	1.4	3.5	3.6	0.7
Disallowed (%)	0.7	0	0	0

**Table 4:** X-ray data collection and model refinement statistics for CBM32-4 and CBM32-6.

*Data collection statistics*	CBM32-4Seleno-Methionine	CBM32-4glcNAc-α-1,4-gal	CBM32-6Seleno-Methionine
Wavelength	0.9796	1.5418	0.9790
Beamline	NSLS X8C	MM-002	SSRL BL9-2
Space group	P4_3_2_1_2	P2_1_2_1_2	P4_3_
Resolution	20.00-1.55 (1.64-1.55)	20.00-2.80 (2.87-2.80)	30.00-1.55 (1.63-1.55)
Cell dimensionα, β, γ (Å)	53.20, 53.20, 110.6090.0, 90.0, 90.0	90.0, 90.0, 90.089.71, 49.89, 63.17	90.0, 90.0, 90.048.81, 48.81, 98.18
*R_merge_*	0.107 (0.403)	0.143 (0.329)	0.048 (0.377)
Completeness (%)	99.9 (99.8)	93.4 (90.5)	100.0 (100.0)
*<I/σI>*	18.9 (7.6)	5.6 (2.5)	30.6 (7.6)
Redundancy	16.3 (16.6)	3.6 (3.8)	15.2 (15.2)
Total reflections	389006	25312	505862
Unique reflections	23846	6965	33325
*Refinement statistics*			
*R* (%)	12.8	28.6	19.6
*R_free_ (%)*	17.3	31.8	23.9
RMSD			
Bond lengths (Å)	0.018	0.006	0.011
Bond angles (°)	1.649	1.053	1.294
Average *B*-factors (Å^2^)			
Protein Chain A	11.1	24.9	22.7
Protein Chain B	N/A	19.6	29.6
Water molecules	30.7	23.7	32.5
Ligand	N/A	69.5 (A); 33.1 (B)	N/A
Number of atoms			
Protein atoms Chain A	1064	1097	1017
Protein atoms Chain B	N/A	1103	982
Water molecules	261	111	174
Ligand	N/A	52	N/A
Ramachandran statistics			
Most favored (%)	95.7	92.6	96.9
Additional allowed (%)	4.3	6.3	1.3
Disallowed (%)	0	1.1	1.8

The structures of CBM32-4 and CBM32-6 were solved by single-anomalous dispersion (SAD) experiments optimized for selenium (see Table 4 for wavelengths at which SAD data were collected). The heavy atom substructures were determined from the SAD data using the program ShelXC/D, while phasing was performed using ShelxE [39]. CBM32-4 crystallized with a single molecule in the AU; three of its potential four selenium sites were found and used for phasing. CBM32-6 crystallized with a two molecules in the AU with each monomer having two potential selenium sites; only one selenium site per monomer was found and used for phasing. Density modification with the program DM [40], [41] was used to improve the phases prior to model building. ARP/wARP [42] was able to build almost complete models, which were completed by manual model building with COOT [43]. Structural refinement of CBM32-6 (selenium derivative) was performed with PHENIX [44] refine using simulated annealing interspersed with manual building in COOT [43]. REFMAC [45] was used to refine CBM32-4. The structure of CBM32-4 in complex with GlcNAc-α-1,4-Gal was solved by molecular replacement using PHASER [46] to find the two molecules in the asymmetric unit. The model was completed by manual building with COOT and refinement with REFMAC; TLS parameters were included in the final refinement cycles of this structure.

The structure of CBM32-5 in complex with galactose was solved by molecular replacement using CpCBM32C from CpGH84C as a search model (PDB id 2j1e [27]) and MOLREP [47] to find the single molecule in the asymmetric unit. Automated model building was carried out with ARP/wARP followed by manual completion with COOT. This structure was used as a starting point to solve the structures of CBM32-5 in complex with other sugars. All refinements were carried out using REFMAC.

In all cases, waters were added using COOT:FINDWATERS. In all datasets 5% of the observations were flagged as “free” and used to monitor refinement progress. Final models were validated with MOLPROBITY [48]. Tables 3 and 4 show the data collection, refinement and final model validation statistics.

### Modeling the CBM32-5 tetrasaccharide complex

A 50 ns molecular dynamics (MD) simulation of the tetrasaccharide, GalNAcα1-4(Fucα1-2)Galβ1-4GlcNAcβ with a reducing terminal methyl, was performed using the pmemd module of the AMBER11 software package [49]. The GLYCAM06g [50] force field was used for the tetrasaccharide parameters while the initial geometry was obtained from the GLYCAM carbohydrate 3D structure web tool [51]. The tetrasaccharide was explicitly solvated with 1724 TIP3P waters [52] and no ions. Minimization was performed for 20,000 steps, half of which used the conjugate gradient method followed by the steepest descent method. A 10.050 ns constant pressure MD (NPT) was used to ensure water and glycan equilibration in which the first 50 ps were used to heat the system from 5 K to 300 K. The final frame from equilibration was used to start the 50 ns NPT production simulation of the tetrasaccharide. In all tetrasaccharide simulations an 8.0 Å van der Waals cutoff was employed, particle mesh Ewald summation (PME)[53] was used for long range electrostatics, 1,4-scaling factors were set to unity, and a dielectric of 1.0 were employed. The Berendsen thermostat was used with a coupling constant of 1.0 ps. Pressure was maintained at 1 atm with a relaxation time of 0.1 ps. The SHAKE [54] algorithm was used to restrain the bonds to hydrogens reducing the time between steps to 2 fs. Production frames were collected at every ps and only the production run was used for further analyses.

The crystal structure of GalNAc-β-Serine bound to the CBM32-5 was used as a template for modeling the tetrasaccharide onto the complex. The GalNAc-β from the template crystal structure and the non-reducing terminal GalNAc-β from the MD simulation were aligned on the ring atoms (C1, C2, C3, C4, C5 and O5) using the alignment algorithm in VMD [55]. Then the MD trajectory of the entire tetrasaccharide was combined together with the template protein coordinates resulting in 50,000 snapshots of the solution tetrasaccharide bound to the crystal protein coordinates. Clashes were removed using a 2,000 step minimization, half conjugate gradient and half steepest descent, for each of the 50,000 complexes where the FF99SB force field [56] was used for the protein. The modified Onufriev, Bashford and Case generalized Borne implicit solvent was used [57] to approximate solvent effects in minimization. All minimizations in developing the CBM-tetrasaccharide complexes used mixed 1,4-scaling, which set van der Waals and electrostatic scaling factors to 1.2 and 2.0, respectively, for the protein (consistent with FF99SB) and unity for the tetrasaccharide (consistent with GLYCAM06). Additionally, a 12.0 Å long-range van der Waals cutoff was employed with PME being used for long-range electrostatics.

The final net energy (including GB solvation contributions) of the CBM-tetrasaccharide complex was used to identify complexes within 15 kcal/mol of the lowest energy complex. This resulted in the selection of 42 complexes, which were further minimized using 10,000 steps of conjugate gradient and 10,000 steps of steepest descent minimization. These new complexes were then ranked according to their overall system energy and grouped together using a 1.0 Å cutoff in root mean squared deviation of the heavy atoms. The models were grouped such that reference structures were selected starting from the lowest energy and ending at the highest energy models. Structures grouped from the lowest energy clusters were excluded from subsequent root mean square deviation grouping analyses meaning any single representation could only belong to one group. Ten clusters were identified in which 60% of the complexes were in the two lowest energy groupings, 33% in the lowest energy group. Energy decomposition was performed on these ten clusters using the MMGBSA.py application in AMBER using the same implicit solvent model as in the minimizations.

### Homology modeling of CBM32-3 and CBM32-2

Structural models of CBM32-3 and CBM32-2 were prepared using the one-to-one threading function of the Phyre2 server [58]. In both cases, the 1.55 Å resolution structure of CBM32-4 was used as a template.

### Accession Codes

Coordinates and structure factors have been deposited in the protein data bank with the following accession codes: **4a3z** for CBM32-4 (seleno-methionine labeled), **4a6o** for CBM32-4 in complex with GlcNAc-α-1,4-Gal, **4a41** for CBM32-5 in complex with galactose, **4aax** for CBM32-5 in complex with GalNAc, **4a45** for CBM32-5 in complex with GalNAc-β-1,3-Gal, **4a44** for CBM32-5 in complex with the Tn Antigen, and **4a42** for CBM32-6 (seleno-methionine labeled).

## Supporting Information

Figure S1 **The energy decomposition profiles of residues within 5.0 Å of the tetrasaccharide, GalNAc-α-1,4(Fuc-α-1,2)-Gal-β-1,4-GlcNAc, modeled onto the crystal structure of CBM32-5.** The non-polar contributions (top), polar contributions (middle), and net binding contributions (bottom) are shown on a per-residue basis. While the predominant interaction is between the protein and GalNAc, the fucose adds significant non-polar contributions to the binding through residues Y1395 and N1396.
(DOC)
