# Supplementary material for: Carbohydrate Recognition by an Architecturally Complex α-N-Acetylglucosaminidase from Clostridium perfringens
Source: PLoS One. 2012 Mar 27;7(3):e33524. doi: 10.1371/journal.pone.0033524 (PMC3313936; doi:10.1371/journal.pone.0033524)

**Supplementary material for:**

**Carbohydrate recognition by an architecturally complex α-*N*-acetylglucosaminidase from *Clostridium perfringens*.**

Elizabeth Ficko-Blean^1^, Christopher P. Stuart^1^, Michael D. Suits^1^, Melissa Cid^1^, Matthew Tessier^2^, Robert J. Woods^2,3^, and Alisdair B. Boraston^1^*

^1^Biochemistry & Microbiology, University of Victoria, Victoria, British Columbia, Canada.

^2^Complex Carbohydrate Research Center, University of Georgia, Athens, Georgia, USA

^3^School of Chemistry, National University of Ireland, Galway, Ireland

*Correspondence should be addressed to: Alisdair B. Boraston, Biochemistry & Microbiology, University of Victoria, PO Box 3055 STN CSC, Victoria, BC, V8W 3P6, Canada. Tel: 250.472.4168. Fax: 250.721.8855. Email: boraston@uvic.ca

Figure S1


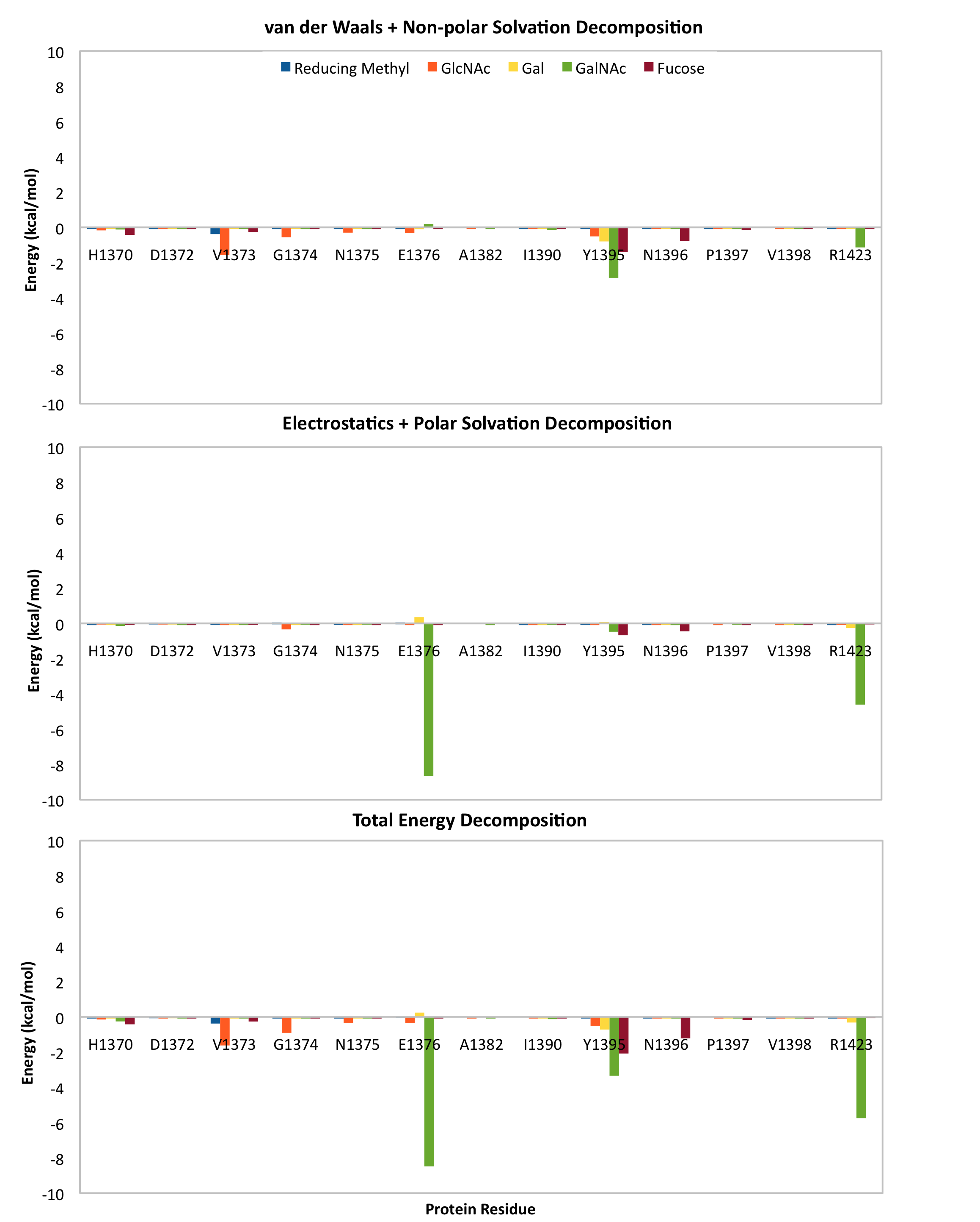

Supplement: Figure S1 — The energy decomposition profiles of residues within 5.0 Å of the tetrasaccharide, GalNAc-α-1,4(Fuc-α-1,2)-Gal-β-1,4-GlcNAc, modeled onto the crystal structure of CBM32-5. The non-polar contributions (top), polar contributions (middle), and net binding contributions (bottom) are shown on a per-residue basis. While the predominant interaction is between the protein and GalNAc, the fucose adds significant non-polar contributions to the binding through residues Y1395 and N1396. (DOC) [file pone.0033524.s001.doc]
